# Clinical implementation of an oncology‐specific family health history risk assessment tool

**DOI:** 10.1186/s13053-021-00177-y

**Published:** 2021-03-20

**Authors:** Si Ming Fung, R. Ryanne Wu, Rachel A. Myers, Jasper Goh, Geoffrey S. Ginsburg, David Matchar, Lori A. Orlando, Joanne Ngeow

**Affiliations:** 1 Cancer Genetics Service, Division of Medical Oncology, National Cancer Centre Singapore, Singapore, Singapore; 2 Centre for Applied Genomics and Precision Medicine, Department of Medicine, Duke University School of Medicine, 304 Research Dr. Box 90141, Office 264, North Carolina 27708 Durham, USA; 3 Department of Medicine, Duke University School of Medicine, 304 Research Dr. Box 90141, Office 264, North Carolina 27708 Durham, USA; 4 Program in Health Services and Systems Research, Duke-NUS Medical School, Singapore, Singapore; 5 Lee Kong Chian School of Medicine, Nanyang Technological University, Singapore, Singapore

**Keywords:** Genetic counselling, Hereditary cancer syndromes, Family history, Risk assessment, Referral

## Abstract

**Background:**

The presence of hereditary cancer syndromes in cancer patients can have an impact on current clinical care and post-treatment prevention and surveillance measures. Several barriers inhibit identification of hereditary cancer syndromes in routine practice. This paper describes the impact of using a patient-facing family health history risk assessment platform on the identification and referral of breast cancer patients to genetic counselling services.

**Methods:**

This was a hybrid implementation-effectiveness study completed in breast cancer clinics. English-literate patients not previously referred for genetic counselling and/or gone through genetic testing were offered enrollment. Consented participants were provided educational materials on family health history collection, entered their family health history into the platform and completed a satisfaction survey. Upon completion, participants and their clinicians were given personalized risk reports. Chart abstraction was done to identify actions taken by patients, providers and genetic counsellors.

**Results:**

Of 195 patients approached, 102 consented and completed the study (mean age 55.7, 100 % women). Sixty-six (65 %) met guideline criteria for genetic counseling of which 24 (36 %) were referred for genetic counseling. Of those referred, 13 (54 %) participants attended and eight (33 %) completed genetic testing. On multivariate logistic regression, referral was not associated with age, cancer stage, or race but was associated with clinical provider (*p* = 0.041). Most providers (71 %) had higher referral rates during the study compared to prior. The majority of participants found the experience useful (84 %), were more aware of their health risks (83 %), and were likely to recommend using a patient-facing platform to others (69 %).

**Conclusions:**

65 % of patients attending breast cancer clinics in this study are at-risk for hereditary conditions based on current guidelines. Using a patient-facing risk assessment platform enhances the ability to identify these patients systematically and with widespread acceptability and recognized value by patients. As only a third of at-risk participants received referrals for genetic counseling, further understanding barriers to referral is needed to optimize hereditary risk assessment in oncology practices.

**Trial Registration:**

NIH Clinical Trials registry, NCT04639934. Registered Nov 23, 2020 -- Retrospectively registered.

## Background

The value of a precision medicine approach to clinical cancer care is becoming increasingly more evident [1] as advancements in genetic technology for sequencing and detection of somatic and germline mutations improve cancer treatment and prevention [2, 3]. Given that 5 to 10 % of cancer cases are hereditary, [4, 5] it is extremely important that patients with potential hereditary cancer syndromes are identified and offered genetic testing so that patients and their family members are aware of treatment options and subsequent steps to mitigate risk of additional cancers [2, 6–9]. For example, poly adenosine diphosphate-ribose polymerase (PARP) inhibitors are used to treat *BRCA*-associated advanced breast and ovarian cancers and are being evaluated for other cancer types [1, 10–12]. When considering surveillance and preventative measures following cancer treatment, those known to have a hereditary cancer syndrome have a wider range of potential measures that should be considered [13, 14].

However, barriers exist preventing widespread and effective adoption of systematic hereditary cancer screening in oncology [15, 16]. Clinicians may be unfamiliar with clinical guidelines on when to refer patients for genetic counselling (GC), resulting in over and underutilization of cancer genetic services [15, 16]. Although family health history (FHH) is essential for identifying at-risk individuals, [17–19] detailed FHH is rarely available in medical records and even less frequently available as structured data that providers can retrieve computationally [16, 20–22]. Hence, FHH collection relies largely on provider ascertainment during the clinical visit [19] but patients are frequently unprepared to provide FHH, partly due to lack of communication among family members and/or lack of awareness of its importance [23]. Due to time constraints and lack of standardization and awareness, clinical providers may have difficulties maximizing the utility of FHH in their clinical practice to identify at-risk individuals [24].

Patient-facing FHH tools have been shown to be better than the current practice of FHH collection by clinicians [19, 25–29] and comparable to FHH collected by genetic counsellors [25]. Currently there are several software programs designed to facilitate identification of at-risk individuals; however, all prior evaluations were done within a primary care context and not one specific to oncology and the majority have not looked at clinical impact beyond risk identification [30].

MeTree, a web-based patient-facing FHH collection software with integrated clinical decision support (CDS), is one such platform [31]. MeTree has been extensively studied in primary care where it has been shown to increase risk identification and impact clinical care [32–34]. We sought to evaluate how MeTree might be used to meet the needs of an oncology setting. After adapting the platform based on feedback from oncology and genetic counsellor study team members, we designed a pilot study to examine its impact in an oncology setting. This paper presents the primary implementation outcomes of risk identification and clinical referral impact.

## Methods

### Overview and study design

This was a hybrid type III implementation-effectiveness study [35] conducted at the breast oncology clinics of two hospitals in Singapore.

### Recruitment and enrollment

All seven clinical providers (six medical oncologists and one breast surgeon) approached agreed to participate in the study. Eligible patients of these providers were offered enrollment face-to-face at their clinic appointments. The inclusion criteria were English-speaking patients with a histologically confirmed breast cancer who had not been referred for GC and/or testing. Written informed consent was obtained from the participants at recruitment.

### Intervention

The MeTree risk assessment platform collects data on 128 medical conditions, including 32 cancers and 22 hereditary cancer syndromes, and analyzes seven cancer risk calculators (BRCAPro, Gail, MMRPro, Tyrer-Cuzick, PREMM, and NCI’s colon cancer risk calculator). MeTree provides CDS for 45 hereditary cancer syndromes and familial cancers in real-time via two reports: one for patients to highlight the high-risk aspects of their personal and FHH and points for discussion with their physician, and one for physicians that indicates the guideline recommendation and high-risk features that triggered the recommendation. Details regarding MeTree’s initial development and validation have been published [31] Since it was initially developed for integration into U.S. primary care practices, study investigators and cancer clinic providers (clinicians and genetic counsellors), recommended several adaptations for oncology practices in Singapore (e.g. including Manchester risk score in decision support recommendations according to American College of Medical Genetics practice guidelines) [17, 36].

At study enrollment, participants were provided with: (1) Information about the importance of FHH, and (2) FHH worksheet listing relative categories (e.g. siblings, aunts, uncles) with a description of the conditions collected in the risk assessment platform to facilitate FHH collection from relatives before entering their information into the software. Participants were scheduled to enter relevant personal history and FHH information into the risk assessment platform one-hour before their next clinic appointment using a study-provided electronic device. Educational resources were available within the platform and a coordinator was available to assist participants if needed. Both patient and provider reports were generated in real-time and reports were given to participants and providers. It was left up to the participant and their clinical provider whether to act on the recommendations provided.

Immediately post-risk assessment, participants completed a survey to assess their experience.

### Measures and outcomes

Data were analyzed from two sources: data entered into and generated by the risk assessment platform and data from the electronic medical record (EMR). Platform data included: FHH pedigree data (including: number of relatives, % of relatives with cancer diagnoses, age of disease onset), Manchester risk scores, clinical decision support recommendations, and triggers for GC recommendations. Pedigrees were assessed for (1) pedigree size, (2) the % of relatives marked as having an unknown FHH, and (3) the % of relatives with cancer history, (4) the % of relatives with cancer for whom age of onset was reported. The quality of the data was assessed by measuring the % of relatives marked as having an unknown FHH and the % of relatives with cancer for whom age of onset was reported. EMR data included: participant demographics, breast cancer details (age of diagnosis, years since breast cancer diagnosis, breast cancer stage, breast cancer type), GC referral status, and genetic testing results. GC referral status and genetic testing results were abstracted six months post-risk assessment. The primary outcomes were: (1) % with a *GC recommendation* by risk assessment, and (2) among those with a recommendation the % *referred to a genetic counsellor* by the clinical provider.

Secondary outcomes include participant responses in the post-risk assessment survey. The survey included 11 items with two being Likert scale items and the remainder yes/no categorical items. The survey was adapted from a previous study [37] and assessed the following areas: participant satisfaction, likelihood to recommend risk assessment platform to others, user experience of platform, preparedness of participants, overall experience and benefits from risk assessment platform.

### Statistical analysis

Participant characteristics, risk assessment results, and FHH data were summarized using counts and percentages for categorical variables or means and standard deviations (SD) for continuous variables. Receipt of a GC recommendation (“GC recommendation” vs. “no GC recommendation”) was examined for differences based on (1) participants’ demographics, (2) FHH using Pearson’s chi-square test for categorical variables, t-test for continuous participant characteristic variables, and Wilcoxon rank-sum test for FHH parameters (pedigree size, % relatives with unknown FHH, % relatives with cancer history, % relatives with cancer for whom age of cancer diagnosis was reported). Among the subset of participants with a GC recommendation, receiving a GC referral (“GC referral” vs. “no GC referral”) was examined for variation based on (1) participant demographics, (2) triggers for GC recommendation, and (3) provider, using Pearson’s chi-square test for categorical variables and t-test for continuous variables. To test for differences in participant feedback on the post-risk assessment survey we analyzed survey responses by (1) FHH parameters using the Kurskal-Wallis test for Likert scale questions (satisfaction and likelihood to recommend) and Wilcoxon rank-sum test was used for the yes/no questions, and (2) GC recommendation using Pearson’s chi-square test. Multivariate logistic regression fixed and mixed effect models were used to evaluate associations of participant demographics with GC recommendation and GC referral status of participants. All statistical analysis was conducted using R statistical software, generalized linear mixed effect models were estimated using the R package lme4.

## Results

### Characteristics of participants

Of the 195 patients approached in breast oncology clinics from 7 January 2019 to 16 August 2019, 121 (62.1 %) consented to participate in the study and 102 completed the study (84.3 % of consented participants). Table 1 outlines key demographics of patients approached, consented and that completed the risk assessment. Among them, patients did not differ significantly in age, race, or breast cancer stage. Age of completed participants ranged from 39 to 81 years old; The racial distribution of completed participants was reflective of the Singapore population [38]. Breast cancer stage was normally distributed with 45.1 % at Stage II. The mean age of diagnosis of completed participants was 52.5 years old, which is comparable to the median age of diagnosis in Singapore (53 years old) [39].

**Table 1:** Characteristics of patients approached, patients consented and completed participants

Characteristics		Patients approached (*n* = 195)		Participants consented (*n* = 121)	Participants completed risk assessment (*n* = 102)		*p*-value^a^
Mean age at BC diagnosis (SD)		52.9 (9.1)		53.1 (9.2)	52.5 (9.2)		0.891
Mean years from BC diagnosis to date approached (SD)		3.5 (4.2)		3.5 (3.6)	3.2 (3.7)		0.991
Race							0.959
Chinese		129 (66.2)		81 (66.9)	67 (65.7)		
Malay		40 (20.5)		20 (16.5)	17 (16.7)		
Indian		13 (6.7)		8 (6.6)	7 (6.9)		
Others		13 (6.7)		12 (9.9)	11 (10.8)		
Overall Breast Cancer Staging					0.952		
0		5 (2.6)		5 (4.1)	4 (3.9)		
I		32 (16.4)		21 (17.4)	20 (19.6)		
II		74 (37.9)		51 (42.1)	46 (45.1)		
III		25 (12.8)		12 (9.9)	12 (11.8)		
IV		38 (19.5)		19 (15.7)	17 (16.7)		
Unknown		21 (10.8)		15 (12.4)	3 (2.9)		

^a^*p*-values were obtained from one-way ANOVA for continuous variables and Pearson’s chi-square test for categorical variables

*BC* breast cancer

### Risk classification

Sixty-six participants (64.7 %) met guideline criteria for GC recommendation. On univariate analyses, those with a GC recommendation were significantly younger (52.3 vs. 61.9,* p* < 0.001), had a significantly younger age at breast cancer diagnosis (48.5 vs. 59.9, *p* < 0.001), a significantly higher Manchester score (5.7 vs. 1.2, *p* < 0.001), and a significantly longer duration since diagnosis (3.8 vs. 2.0, *p* = 0.004) as compared to those without a GC recommendation. (Table 3) Race, breast cancer stage and assigned clinical provider had no significant impact on receiving a GC recommendation in univariate analyses. In multivariate logistic regression modelling, GC recommendation was associated with younger age (Odds Ratio (OR) -0.17, SE 0.05, *p* < 0.001), having a higher Manchester score (OR 2.13, SE 0.10, *p* < 0.001) and a higher percentage of relatives with cancer history (OR 1.14, SE 0.03, *p* = 0.03) (Table 2).

**Table 2:** Participant characteristics and family health history parameters according to GC recommendation

Characteristics	Completed (*n* = 102)		GC recommendation (*n* = 66)	No GC recommendation (*n* = 36)	*p*-value
Mean age at enrollment^a^ (SD)	55.7 (9.0)		52.3 (7.8)	61.9 (7.7)	< 0.001
Mean age at BC diagnosis^a^ (SD)	52.5 (9.2)		48.5 (7.5)	59.9 (7.2)	< 0.001
Mean years from BC diagnosis to enrollment^a^ (SD)	3.2 (3.7)		3.8 (4.2)	2.0 (2.1)	0.004
Mean Manchester score^a^ (SD)	4.1 (4.3)		5.7 (4.2)	1.2 (2.7)	< 0.001
Mean pedigree size^b,c^ (SD)	10.7 (3.6)		10.7 (3.5)	10.7 (3.6)	0.986
Mean % relatives with unknown history^b^ (SD)	28.9 (19.5)		27.3 (17.7)	31.7 (22.4)	0.390
Mean % relatives with cancer history^b^ (SD)	15.2 (13.6)		16.9 (14.2)	12.0 (11.9)	0.093
Mean % relatives with age of cancer diagnosis reported^b,d^ (SD)	93.2 (16.7)		91.9 (17.5)	96.0 (14.6)	0.246
Race^a^					0.425
Chinese	67 (65.7)		45 (68.2)	22 (61.1)	
Malay	17 (16.7)		8 (12.1)	9 (25.0)	
Indian	7 (6.9)		5 (7.6)	2 (5.6)	
Others	11 (10.8)		8 (12.1)	3 (8.3)	
Breast Cancer stage^a^					0.760
0		4 (3.9)	3 (4.5)	1 (2.8)	
I		20 (19.6)	15 (22.7)	5 (13.9)	
II		46 (45.1)	28 (42.4)	18 (50.0)	
III		12 (11.8)	7 (10.6)	5 (13.9)	
IV		17 (16.7)	12 (18.2)	5 (13.9)	
Unknown		3 (2.9)	1 (1.5)	2 (5.6)	
GC referral from clinical provider^a^					< 0.001
Yes		24 (23.5)	24 (36.4)	0	
No		78 (76.5)	42 (63.6)	36 (100.0)	
Clinical provider^a^					0.902
Provider A		38 (37.3)	22 (33.3)	16 (44.4)	
Provider B		36 (35.3)	25 (37.9)	11 (30.6)	
Provider C		8 (7.8)	6 (9.1)	2 (5.6)	
Provider D		7 (6.9)	4 (6.1)	3 (8.3)	
Provider E		7 (6.9)	5 (7.6)	2 (5.6)	
Provider F		3 (2.9)	2 (3.0)	1 (2.8)	
Provider G		3 (2.9)	2 (3.0)	1 (2.8)	

*GC* Genetic Counselling, *SD* Standard Deviation, *BC* Breast cancer

*(%) refers to the proportion of participants among those with GC referral

^a^*p*-values were obtained from Pearson’s chi-square test for categorical variables and independent-

samples t-test for continuous variables (*p* < 0.05)

^b^*p*-values were obtained from Wilcoxon-rank sum test as FHH parameters not normally distributed (*p* < 0.05)

^c^ Excluding the participant

^d^ Restricted to families with at least one relative with cancer, excluding cancer types without age of diagnosis reported. If relative has more than 1 cancer type reported, age is reported for at least one of those

**Table 3:** Associations between participant characteristics and GC recommendation from multivariate logistic regression

Characteristics	GC recommendation (*n* = 66) OR (95 % CI)^a^	*p*-value
Age at enrollment Mean (95 % CI)	-0.17 (-0.26, -0.08)	0.001
Manchester score	0.35 (0.15, 0.55)	< 0.001
% relatives with cancer history	0.06 (0.01, 0.11)	0.017
Race		
Malay	-1.17 (-2.90, 0.56)	0.189
Indian	0.74 (-1.92, 3.40)	0.584
Others	-0.42 (-2.50, 1.66)	0.693
Chinese	Ref	
Overall BC staging		
0	Ref	
I	0.67 (-4.55, 5.89)	0.801
II	-0.53 (-5.55, 4.49)	0.836
III	0.70 (-4.43, 5.84)	0.788
IV	0.17 (-4.95, 5.28)	0.950

*GC* Genetic Counselling, *OR* Odds Ratio, *CI* Confidence Interval, *BC* Breast Cancer.^a^ Values are odds ratio with 95 % CI from multivariate logistic regression analysis.

### Risk evaluation

**Fig. 1:**
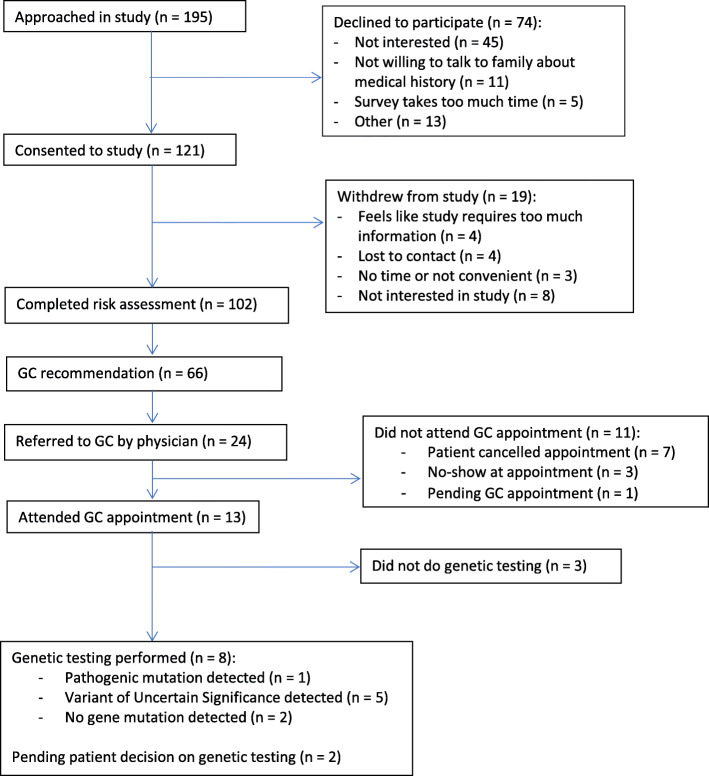
Flowchart of participants in study

Of the 66 participants with a GC recommendation, twenty-four (36.4 %) were referred for GC by their clinical providers. Age at enrollment, years since their breast cancer diagnosis, race, and breast cancer stage had no impact on GC referral. Some clinical providers were more likely to refer at-risk participants to GC than others (*p* = 0.04) (Tables 4 and 5). Among those referred, 13 (54.2 %) attended their GC appointments and eight (61.5 % of those who attended) underwent genetic testing. Of those tested, one had a pathogenic variant, five had Variants of Uncertain Significance (VUS), and two had benign variants. (Fig. 1)

**Table 4:** Participant characteristics by GC referral status among participants with a GC recommendation

Characteristics	GC referral (%) (*n* = 24)	No GC referral (%) (*n* = 42)	*p*-value^a^
Mean age at enrollment (SD)	51.5 (9.5)	52.7 (6.7)	0.563
Mean age at diagnosis (SD)	47.8 (8.8)	48.9 (6.7)	0.626
Mean years from diagnosis to enrollment (SD)	3.6 (4.8)	3.9 (3.8)	0.825
Race			0.707
Chinese	15 (62.5)	30 (71.4)	
Malay	3 (12.5)	5 (11.9)	
Indian	3 (12.5)	2 (4.8)	
Others	3 (12.5)	5 (11.9)	
Breast Cancer stage			0.488
0	2 (8.3)	1 (2.4)	
I	6 (25.0)	10 (21.4)	
II	10 (41.7)	18 (42.9)	
III	3 (12.5)	4 (9.5)	
IV	2 (8.3)	10 (23.8)	
Unknown	1 (4.2)	0	
Clinical provider			0.036
Provider A	13 (54.2)	9 (21.4)	
Provider B	3 (12.5)	22 (52.4)	
Provider C	3 (12.5)	3 (7.1)	
Provider D	2 (8.3)	2 (4.8)	
Provider E	2 (8.3)	3 (7.1)	
Provider F	0	2 (4.8)	
Provider G	1 (4.2)	1 (2.4)	

*GC* Genetic Counselling, *CI* Confidence Interval

^a^*p*-values were obtained from Pearson’s chi-square test for categorical variables or independent samples t-test for continuous variables (*p* < 0.05)

**Table 5:** Associations between participant characteristics and GC referral among those with a GC recommendation from multivariate logistic regression

Characteristics	GC referral (*n* = 24) OR (95 % CI)^a^	*p*-value
Age at enrollment Mean (95 % CI)	-0.02 (-0.10, 0.05)	0.546
Race		
Malay	-0.10 (-1.94, 1.75)	0.919
Indian	1.46 (-0.56, 3.49)	0.176
Others	-0.03 (-1.75, 1.70)	0.976
Chinese	Ref	
Overall BC staging		
0	Ref	
I	-1.03 (-3.89, 1.84)	0.482
II	-1.45 (-4.25, 1.34)	0.308
III	-0.83 (-3.92, 2.27)	0.602
IV	-2.41 (-5.44, 0.62)	0.119

*GC* Genetic Counselling, *OR* Odds Ratio, *CI* Confidence Interval, *BC* Breast Cancer

^a^Values are odds ratio with 95% CI from multivariate logistic regression analysis.

### FHH data entered by participants

Details of FHH entered by participants are provided in Table 3. Within FHH pedigrees, participants listed a mean of 10.7 relatives in their pedigree, a mean of 28.9 % of relatives had unknown FHH and majority of cancer-affected relatives had age of onset reported (93.2 %). Those with GC recommendation had a trend towards higher % relatives with cancer history (Mean: 16.9 % vs. 12.0 %, *p* = 0.09). There was no significant difference in the number of relatives entered or the quality of the FHH data entered (i.e. % relatives with unknown FHH, % relatives with age of cancer diagnosis reported) between those who did and did not receive a GC recommendation.

The clinical justifications for the GC recommendations are listed in Table 6. The most common clinical justification was a personal age of breast cancer diagnosis less than 50 years (*n* = 41/67) and an elevated Manchester risk score (*n* = 13/67). There was no obvious difference in clinical justification for GC recommendation between those who were or were not referred for GC.

**Table 6:** Clinical justification for GC recommendation according to GC referral status

	All with GC recommendation (*n* = 66)	GC referral(*n* = 24)	No GC referral (*n* = 42)
*Personal medical history*			
BC diagnosis at age ≤ 50	41	18	23
Bilateral breast cancer	2	2	0
TNBC diagnosis age < 60	3	2	1
Ovarian cancer diagnosis	1	1	0
Endometrial cancer diagnosed at age < 50	1	0	1
*Family history*			
≥ 1 relative with Li-Fraumeni syndrome related cancer + Personal BC diagnosis at age ≤ 45	9	1	8
≥ 1 relative with Li-Fraumeni syndrome related cancer at age ≤ 45	5	3	2
≥ 3 family members with same cancer	2	0	2
≥ 2 relatives with colorectal cancer	1	0	1
≥ 3 cases of Lynch syndrome related cancer in family	5	1	4
≥ 2 cases of breast, ovarian, pancreatic or prostate cancer in family	5	1	4
≥ 1 FDR with breast cancer at age ≤ 50	3	2	1
≥ 1 FDR with colorectal cancer at age < 50	3	2	1
≥ 1 FDR with ovarian cancer	2	0	2
*Risk score*			
Manchester risk score meets criteria	13	6	7

*GC* Genetic Counselling, *TNBC* Triple Negative Breast Cancer, *FDR *First Degree Relative

### Participant feedback on using a patient‐facing risk assessment platform

In the post-risk assessment survey, the majority of participants reported being very satisfied with the platform (66.7 %) and the majority were likely to recommend it to others (68.6 %) (Table 7). Related to the user experience, most participants found it to be easy to use (86.3 %), the questions did not make most feel anxious (88.2 %), and almost all found the questions easy to understand (98.0 %). Regarding preparedness of participants, the majority found the FHH collection worksheet to be helpful (75.5 %). However, most did not talk to relatives before entering FHH data (66.7 %), and the majority did not feel they had enough information about some of their relatives (76.5 %). Regarding overall experience and benefits, 84.3 % found completing the risk assessment platform to be a useful experience while 34.2 % of participants learned a lot about their FHH that they did not know before. The majority of participants reported being more aware of their health risks after completing the risk assessment (83.3 %).

**Table 7:** Participant responses in post-risk assessment satisfaction survey

Questions	Responses	Completed (%) (***n*** = 102)
Q1. Satisfaction with risk assessment platform experience	Mean (95% CI)	3.8 (3.6 – 3.9)
	1 – Very poor	1 (1.0)
	2 – Somewhat unsatisfactory	4 (3.9)
	3 – About average	29 (28.4)
	4 – Very satisfactory	53 (52.0)
	5 - Superior	15 (14.7)
Q2. Likelihood to recommend risk assessment platform to others	Mean (95% CI)	3.9 (3.7 – 4.0)
	1 – Not likely	1 (0.8)
	2 – Unlikely	6 (5.0)
	3 – Somewhat likely	25 (24.5)
	4 – Likely	45 (44.1)
	5 – Very likely	25 (24.5)
The risk assessment platform was easy to use.^a^	Yes	88 (86.3)
	No	7 (6.9)
Answering the questions made me anxious.^a^	Yes	10 (9.8)
	No	90 (88.2)
The questions were easy to understand.^a^	Yes	100 (98.0)
	No	1 (1.0)
The family history worksheet used to help collect information was helpful.^a^	Yes	77 (75.5)
	No	5 (4.9)
I talked with relatives about our family’s health history before using the risk assessment platform.^a^	Yes	32 (31.4)
	No	68 (66.7)
I didn’t have enough information about some people in my family when completing the risk assessment platform.^a^	Yes	78 (76.5)
	No	22 (21.6)
Completing the risk assessment platform was a useful experience.^a^	Yes	86 (84.3)
	No	5 (4.9)
I learned a lot about my family’s health history that I did not know.^a^	Yes	41 (34.2)
	No	45 (44.1)
I am more aware of my health risks.^a^	Yes	85 (83.3)
	No	9 (8.8)

^a^Percentages of participants that responded ‘don’t know’ were not reflected in this table.

Participants were more likely to rate the risk assessment platform as easy to use if they reported a lower proportion of relatives with unknown history (26.1 % unknown history in those who reported the risk assessment as easy to use vs. 49.5 % unknown history in those who found the risk assessment difficult, p = 0.01). Those with a lower proportion of relatives with cancer and those who did not receive a GC recommendation reported greater satisfaction with the risk assessment platform (p = 0.002 and p < 0.001 respectively).

## Discussion

Our study evaluated the impact of a web-based patient-facing FHH risk assessment platform on identifying breast cancer patients with potential hereditary cancer syndromes and its effects on care delivery. This study demonstrates that patient-facing FHH risk assessment platforms can be implemented into oncology settings, with strong patient support. We found that a significant proportion of breast cancer study participants are at-risk for hereditary cancer syndromes. Yet, only one-third were referred for GC by their providers and only half of those referred attended the GC session. Of those who attended, 62 % completed testing and a large majority of those tested were found with a VUS or pathogenic variant. These findings are similar to what has been observed when implementing standardized risk assessment within the primary care environment. In those studies, a significant proportion of the population was found to be at risk for hereditary cancer syndromes [27, 30, 34, 40] (though lower than in a population pre-selected for having breast cancer as in this study) but as in this study, while there is some uptake of risk recommendations, there remain challenges to be addressed to improve clinical impact [32, 41].

### FHH entered and participant feedback on risk assessment

Overall the quality of the FHH entered by participants was good. Those with a GC recommendation trended towards a higher proportion of relatives with cancer history, were younger, and had higher Manchester scores. This demonstrates that the risk assessment platform was performing as intended as risk algorithms were based on current practice guidelines, which recommend breast cancer patients diagnosed at a young age and/or with family members with certain cancer characteristics to undergo GC [17]. Those found to be at risk did not differ in race, assigned clinical provider, or quality of FHH data entered, which supports a lack of bias in the tool and that risk identification was not simply due to bias towards participants knowing their FHH better than those not found to be at risk.

Participant feedback on using the risk assessment platform showed strong participant acceptance, with the majority reporting a high ease of use, ease of understanding and no anxiety. Despite being provided with educational materials beforehand to facilitate FHH collection, 66.7 % of participants did not talk to relatives about FHH before using the platform and hence, majority did not have enough information about some relatives when completing it. This suggests that other barriers are present, other than lack of materials to facilitate FHH collection, that hinders patients’ preparedness to provide FHH. Despite this, most participants felt they benefited from the risk assessment; they were more aware of their health risks and found it to be useful.

### GC referral from clinical provider

The significant number of previously unidentified at-risk patients meeting practice guidelines for GC in this study demonstrates the need for more systematic and comprehensive FHH risk assessment within current oncology practice. Although it is recommended that clinical providers refer patients for genetic counselling early on in their treatment for timely management,[42, 43] we found that patients with GC recommendation frequently were many years out from their breast cancer diagnoses. Even after identification, barriers remain in the referral process. Of the 67 participants with a GC recommendation, 43 were not referred for GC. There was no obvious difference in clinical justification for GC between those with and without GC referral, but some clinical providers were more likely to refer than others. Rather than clinical factors, the significant proportion of participants that were not referred was likely due to a combination of patient and provider factors. In previous studies, provider barriers to referring patients for genetic services were lack of awareness of risk factors, hereditary conditions and genetic services (including unknown or assumed high costs of genetic testing), inadequate FHH assessment and inadequate referral coordination.[44–46] In order to increase appropriate GC referrals further, more must be done to integrate risk assessment platforms into current workflows and referral processes, and to increase provider awareness of hereditary conditions. While risk assessment was offered systematically to any eligible patient, it was not integrated into the EMR and this lack of integration may have resulted in a reduced impact of risk report results on clinical care. Additional barriers to uptake of GC recommendations by clinicians and participants are being explored through an ongoing qualitative aim of this study. Not unlike other public health measures, real change will require change at health policy levels and systematic implementation with prioritization of proactive care over a traditional reactive care model.

Although not captured systematically, study clinical providers recorded in the EMR for at least four participants that GC referral was discussed after receiving a GC recommendation, but clinical referral was not made due to patient preferences. Additionally, of the 24 patients with a GC referral, only 13 (54.2 %) attended their appointments. In previous studies, patient barriers to GC attendance were cost, emotional concerns, family concerns and low perceived personal relevance [47–49]. The cost of genetic testing for hereditary cancer conditions in Singapore is at present an ”out-of-pocket” [49] expense and has been reported as a barrier to referral and uptake [46, 50]. In a local study, it was found that subsidizing the cost of genetic testing resulted in an increase in uptake rate and could be cost-saving [49]. This suggests that raising awareness of the need for GC among patients may be insufficient and that risk assessment should be paired with interventions targeting specific barriers to GC. Prior intervention studies aiming to increase genetic testing uptake [51–56] by providing participants with more information on genetic testing through various avenues (e.g. educational resources, interactive program) reported intervention participants being more informed about genetic testing with improved knowledge. However, the interventions did not impact significantly the uptake of genetic testing.

Of the referred, 13 (54.2 %) attended their GC appointments and eight (61.5 % of attended) went through genetic testing. The medical and family health history of the three participants that declined testing did not meet the National Comprehensive Cancer Network (NCCN) criteria for *BRCA1/2* testing [57] but testing of other relevant genes was offered by genetic counsellors.

### Limitations

There were limitations to the conclusions which can be drawn from this study. It was a small sample size given that it was a pilot to assess feasibility only. Outcomes may have been impacted by survivor bias as we offered enrollment to any patient with breast cancer history and no prior referral to GC and/or testing, hence, there was a mix of participants with incident and prevalent breast cancer cases. Additionally, excluding those previously tested could have resulted in a bias towards selecting participants with more anxiety towards GC. If evaluating in an unselected population with incident breast cancer, GC referral and testing rates might have been higher. The web-based nature of the FHH risk assessment platform could also have affected the uptake and study progression of individuals with lower comfort with technology although a coordinator was available to assist. Finally, the healthcare setting in which a study is performed has the potential to limit its applicability to other healthcare settings as other countries may have different models of care delivery and financing which impact uptake.

## Conclusions

The implementation of a risk assessment platform in oncology clinics identified a significant proportion of breast cancer patients with previously unidentified hereditary cancer risk and facilitated GC referrals. Further evaluation of the barriers and understanding of how to make risk assessment more accessible to patients and clinical providers is warranted, in order to optimize the use of a systematic risk assessment in oncology clinical practice and improve care.

## Data Availability

The datasets used and/or analyzed during the current study are available from the corresponding author on reasonable request.

## Abbreviations

FHH: Family health history
GC: Genetic counselling
EMR: Electronic medical record
VUS: Variant of uncertain significance
OR: Odds ratio
